# The South African Rea Phela Health Study: A randomized controlled trial of communication retention strategies

**DOI:** 10.1371/journal.pone.0196900

**Published:** 2018-05-24

**Authors:** James M. Rhyne, Alexandra Mumbauer, Paul Rheeder, Megan N. Hall, Jeanine Genkinger, Andrew Medina-Marino

**Affiliations:** 1 School of Health Systems and Public Health, Faculty of Health Sciences, University of Pretoria, Pretoria, South Africa; 2 Foundation for Professional Development, Pretoria, South Africa; 3 Department of Internal Medicine, Faculty of Health Sciences, University of Pretoria, Pretoria, South Africa; 4 Department of Epidemiology, Mailman School of Public Health, Columbia University, New York, New York, United States of America; 5 Herbert Irving Comprehensive Cancer Center, Columbia University, New York, New York, United States of America; University of Washington, UNITED STATES

## Abstract

Epidemiological transitions are occurring throughout Africa. To inform public health programs and policies, longitudinal cohorts investigating non-communicable diseases are needed. However, loss-to-follow up is a major problem. In preparation for a longitudinal study, we conducted a randomized controlled trial to test communication-based retention strategies (message content and delivery methods) among a pilot cohort of South African healthcare workers (n = 1536; median age = 36; women = 1270). Two messaging formats across three delivery modes were tested. Response rates were analyzed by intervention, survey return date and method using chi-square tests and univariate logistic regression. Sixty-seven of 238 (17.4%) control group participants and 238 of 1152 (24.6%) intervention group participants were retained (OR 1.54: CI 1.15–2.07; *P* = 0.004). Odds of being retained were 1.68 times greater for participants who received regular contact and themed messages compared to control (CI 1.22–2.32; *P* = 0.001). Neither health status nor clinical condition affected response rates (*P*>0.05). Time-to-first contact did not impact response rates (*P*>0.05). Message content and delivery method influenced response rates compared to the control, however no difference was found between intervention groups. Although greater retention is required for valid cohort studies, these findings are the first to quantitatively assess retention factors in Africa.

## Introduction

Few large, long-term cohort studies focused on non-communicable diseases (NCDs) have been performed in sub-Saharan Africa (SSA) [1–4]. Unfortunately, such studies are complicated by loss to follow-up of study participants, especially in SSA where under-developed infrastructure, poverty and highly mobile populations may make participant retention particularly challenging [4]. South Africa’s well-developed research infrastructure, unique epidemiological transition, and burden of colliding epidemics of non-communicable and communicable diseases [5] offers a unique environment in which to conduct prospective cohort studies focused on NCDs [1]. However, as participation in cohort studies continues to decline [6], without studies that explicitly test and validate effective retention strategies, the ability to attract funding and justify the allocation of scarce resources for large, prospective cohort studies focused on NCDs remains difficult.

In South Africa, the Birth-to-Twenty (BT20) cohort study, which is focused on child and adolescent health and development [7], observed that using a theme or identity, maintaining regular contact, and cultivating benefit by clearly explaining the potential benefit to participants improved retention (personal communications with BT20 investigators). However, average attrition per year was 4% over eight years [8]. The South African National Institute for Occupational Health (NIOH) observed that registered nurses responded to time-sensitive messages with a clearly stated purpose; however, overall response rates were low, with participants citing they were too busy and suffered from survey fatigue (personal communications with NIOH). Similarly, other professions of HCWs, such as community health workers (CHWs), also suffered from survey fatigue; however, they were more likely to respond if they felt they were contributing to a better society (personal communications with NIOH). Understanding these types of motivations behind behavior for various groups of health workers is a key component of audience segmentation and critical step for determining appropriate messaging. Based on the health belief model of behavior change, individuals assess the proposed benefits of changing behavior before determining a course of action [9]. Knowing what benefits appeal to one group over another can then be used to drive the right message to the right person to elicit change in behavior or encourage response. This strategy also reflects best practices in commercial business communication theory, which stress the importance of knowing the audience.

Although improving retention requires more than messaging content alone, little is known about the optimal delivery methods. According to a 2012 policy paper from Research ICT Africa (RIA), 84.2% of South Africans own a mobile phone [10]. However, a 2014 study by Effective Measure found that there are more subscriber identity module (SIM) cards than people in South Africa [11]. This suggests that there may be limitations to using mobile communications alone to target individuals, as owning more than one SIM card and/or mobile phone can make tracking participants difficult. Alternatively, traditional delivery methods such as postal mail are available, but delivery to informal settlements, townships, or rural areas may be unreliable and variable.

Theories of change in health communication agree that changing behavior is not a one-time event. Change is a process that requires messages to be delivered through multiple channels over sustained periods of time. No one method of delivery can be relied upon to communicate the message. In health communications, delivery tactics known to improve retention in general have included: reminder letters combined with re-sending the survey [12]; multiple strategies and themes, combined with in-person follow-up [13]; community involvement and engagement so participants feel connected [8]; contact tracing and tracking in hard-to-reach populations [8]; and shorter and more regular contact intervals to keep participants engaged [8]. Taking advantage of multiple channels also corresponds to established best marketing practices used to maximize impressions and improve the likelihood of response [14]. However in public health, a review of retention efforts has repeatedly highlighted the lack of rigorous evaluations of cohort retention strategies [13]. What is known is limited to “lessons learned” from studies in the United States (US) and United Kingdom (UK) [12]. Furthermore, while informative, what has worked in the US and the UK may not work in low- and middle-income countries.

To address the gap in retention effectiveness in South Africa, we aimed to: 1) test the impact of messaging content (MC) and delivery method (DM) on retention rates at six-month follow-up; 2) measure retention differences between two types of MC and by four DMs; and 3) measure survey return proportions by method (mail or online) and time (prior to or after contact tracing). We hypothesized that themed messaging would lead to higher retention than generic and that consistent contact using any delivery method would retain more participants than minimal contact.

## Materials and methods

We performed a randomized controlled trial to test the effectiveness of MC and DM on the retention of HCWs enrolled in the Rea Phela Health Study during nationwide, multi-day training sessions conducted by the Foundation for Professional Development (FPD) from March through December 2014. Participants were incentivized to participate by returning one of four randomly assigned health questionnaires before the end of the training event. Completed questionnaires were entered into a drawing to win a R100 (~$9.25) grocery gift card. Participants were asked to provide contact information, including their primary mobile number, work number, postal address, and email address. To encourage provision of more accurate and complete contact details, the introduction presented by FPD training staff emphasized the importance of reaching participants in the future.

Inclusion criteria for the six-month follow-up study included providing: 1) informed consent and agreement to be contacted in six months (obtained as part of the initial questionnaire consenting process); 2) a valid, 10-digit mobile phone number; and; 3) professional title or category for random assignment (medical doctors were excluded due to insufficient numbers to fill a statistically significant intervention arm). Participants were excluded for failure to provide any contact details. Ethical approval was received from the University of Pretoria (UP), Faculty of Health Sciences Ethics Committee, FPD and Columbia University.

**Fig 1:**
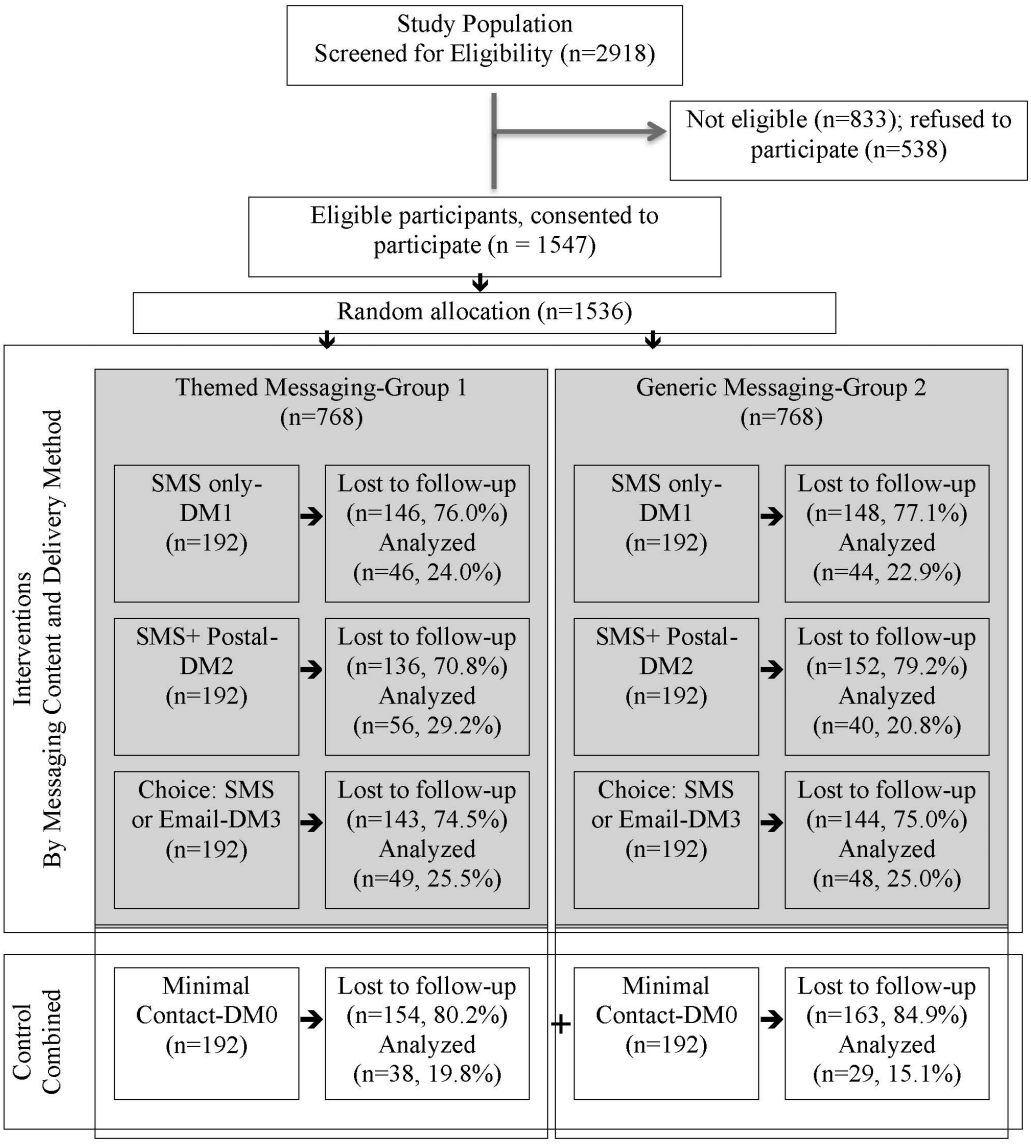
Randomized controlled trial flow diagram for the Rea Phela Health Study.

Intervention strategies were derived from the health belief model and theories of change to design and test two intervention strategies: 1) MC and 2) DM. MC was divided into two groups, themed messaging (Group 1) and generic messaging (Group 2). DM was divided into four groups: i) welcome and survey delivery SMS only (DM0); ii) monthly SMS (DM1); iii) SMS+Postal (DM2); and iv) participant choice of either SMS or email (DM3); intervention activities are described in greater detail below. Operational integration of interventions occurred by stratifying the two MC groups by the four DM groups (Fig 1), resulting in eight sub-groups, randomly assigned using SAS^®^ (Cary, North Carolina, USA). Those in DM3 who did not select their preferred delivery method were assigned to receive SMS because email addresses were not available for everyone. Group 1 and Group 2 participants assigned to DM0 were combined to form the study control group of limited contact without any messaging theme. All other sub-groups were collectively referred to as the interventions. To ensure that no differences existed between Group 1 and Group 2, we compared both groups by sex, age, province and profession. Bias was minimized through random sequence generation, allocation concealment, blinding of participants and personnel, anonymous response assessment and reporting. Because interventions were delivered via text, email and postal mail, participants were not aware of anyone else in the study or aware of other types of messaging or message formats.

Sample size was calculated using the following assumptions: 95% confidence interval (CI); 80% power; ability to detect a difference in retention of 10% between groups [15]. An additional 10% was added to account for attrition, resulting in a total sample size of 192 per sub-group.

### Interventions

Intervention participants were contacted once per month starting October 2014 through delivery of the follow-up questionnaire in April 2015. To start, all participants received a welcome SMS message. Opt-outs were removed from further interventions, except final survey delivery. The control group only received two messages, 1) a welcome SMS and 2) a postal letter, prior to the delivery of their follow-up survey.

**Fig 2:**
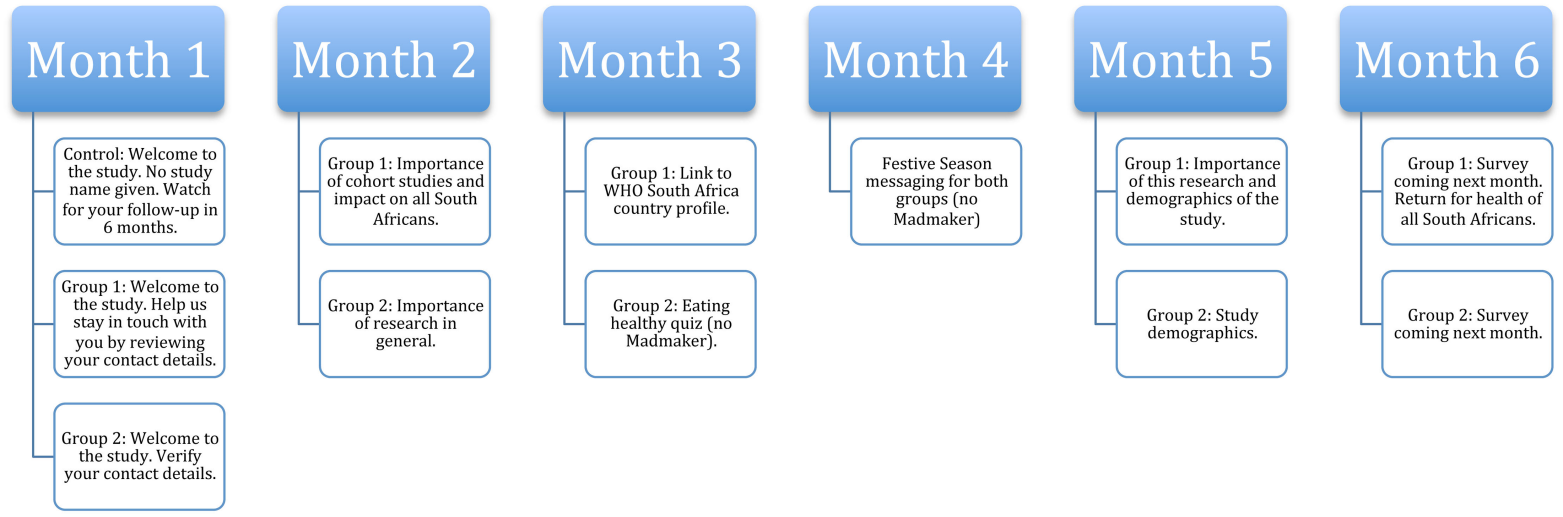
Messaging content by month and group.

Intervention messaging featured the use of Madmaker, a mobile campaign communication tool, which extended the utility of SMS by linking to a (dot) mobi site, allowing extra messaging and branding to be conveyed on any feature phone or smartphone. In the welcome SMS, intervention participants received a link to a Madmaker with a request to confirm their contact details and supply the names and mobile phone numbers of two additional contacts. Successive messaging content for the intervention groups included Madmakers, except where noted (Fig 2). Content also included links to the study Facebook page, website and online health quizzes. In addition, content highlighted study identity for the proposed Rea Phela Health Study; the name Rea Phela, loosely translated as “we are healthy,” and which was developed using focus group discussions with Zulu- and Sotho-speaking FPD employees.

For consistency, all SMS and emails were sent on a Tuesday afternoon between 15:00 and 15:30 South African Standard Time. Emails and letters were “signed” by Ntombi, a fictitious study representative. Postal mail letters were mailed the same day. Participants were able to communicate with study staff via SMS or email; no phone number was given.

The six-month follow-up questionnaire (S1 Appendix) was mailed to all participants with a letter, a pre-printed questionnaire number, a postage-paid envelope and a pencil. Control group participants received a generic letter without the Rea Phela study name. Intervention groups 1 and 2 received letters matching content themes noted in Fig 2. After the questionnaire was mailed, intervention participants were sent a reminder SMS which included the return due date and a Madmaker link to an online version of the survey developed using Qualtrics (Provo, Utah, USA). If the participant had already returned the questionnaire via mail, they were told to ignore the message, otherwise they could choose to complete the online questionnaire. Two more sets of reminder messages were sent via SMS with a direct link to the survey and a new deadline. Controls did not receive reminder messaging or the option to complete the survey online.

Contact tracing of those intervention participants who did not return their survey by the stated deadline was conducted over two-weeks in June 2015; a maximum of two attempts was made using the mobile number provided. If the participant was not reached, and had still not responded to the questionnaire, the participant was considered lost to follow up. Contact tracers recorded whether contact was made and if the participant reported already returning the questionnaire or intent to return it if not yet completed. This information was linked back to the study database using the participants unique study number and used to track return of the questionnaires.

### Data collection, management and statistical analysis

Participant personal contact information (name, surname, email address, postal address, and mobile number) and socio-demographic information (age, sex, province, and profession) were extracted from the initial HCW study. Updated participant contact information received via the study website, email, or Madmaker was sorted and linked to existing participant data via primary mobile phone number, name or email address. The unique, pre-printed number assigned to each paper questionnaire was used to link return information with participant identification numbers (PIDs). Upon return of the questionnaire, date of return, method of return (mail or online), and date of survey completion (if known) were recorded.

Results were analyzed using intent-to-treat analysis [16]. Results were tabulated using frequencies and percentages for categorical variables, and further stratified by age, sex, profession and province. Retention rates were calculated by dividing the number of responses by the total in the group, and further described via stratification by how (paper/mail or electronic/online) and when (prior to or after contact tracing) returned. Chi-square tests and univariate logistic regression analyses were used to perform pair-wise comparisons in retention rates between intervention and control groups, as well as between each delivery method and messaging content group. Wald tests were used to test for interaction between MC and DM. A *P*-value of < 0.05 was considered statistically significant; however, *P*-values were adjusted and noted where applicable for multiple comparisons, using the Bonferroni method [17]. All statistical analysis were performed using STATA^®^, version 14 (College Station, Texas, USA).

## Results

No differences in socio-demographic characteristics were found between Group 1 and Group 2 after randomization (Table 1). Sub-group randomization also showed no difference in socio-demographic characteristics (data not shown).

**Table 1:** Comparison of socio-demographic characteristics between Group 1 and Group 2.

Socio-Demographics	Intent-to-Treat^a^ Participants (n = 1536)	Randomization^b^		*P*-value
		Group 1 (n = 768)	Group 2 (n = 768)	
Age (Median)	36 (17–65)	37 (17–64)	36 (17–65)	> 0.99†
< 24	199 (13.1)	105 (13.8)	94 (12.3)	
25–34	479 (31.4)	230 (30.2)	249 (32.6)	
35–44	449 (29.4)	227 (29.8)	222 (29.1)	
45–54	309 (20.3)	157 (20.6)	152 (19.9)	
> 55	89 (5.8)	43 (5.6)	46 (6.0)	
Sex				0.18††
Female	1270 (82.7)	623 (81.1)	647 (84.2)	
Male	207 (13.5)	112 (14.6)	95 (12.4)	
Province				0.48††
Eastern Cape	17 (1.1)	10 (1.3)	7 (0.91)	
Free State	44 (2.9)	27 (3.5)	17 (2.2)	
Gauteng	704 (45.8)	346 (45.1)	358 (46.6)	
Kwazulu-Natal	1 (0.07)	1 (0.13)	0 (0.0)	
Limpopo	463 (30.1)	219 (28.5)	244 (31.8)	
Mpumalanga	231 (15.0)	123 (16.0)	108 (14.1)	
Northern Cape	31 (2.0)	15 (2.0)	16 (2.1)	
North West	6 (0.39)	4 (0.52)	2 (0.26)	
Western Cape	17 (1.1)	10 (1.3)	7 (0.91)	
Profession				0.68††
Registered Nurse	394 (25.7)	199 (25.9)	195 (25.4)	
Enrolled Nurse	126 (8.2)	61 (7.9)	65 (8.5)	
Auxiliary Nurse	79 (5.1)	39 (5.1)	40 (5.2)	
Lay Counselor	148 (9.6)	73 (9.5)	75 (9.8)	
Community Health Worker	404 (26.3)	203 (26.4)	201 (26.2)	
Allied Health Worker	21 (1.4)	12 (1.6)	9 (1.2)	
Educator/Trainer	51 (3.3)	28 (3.6)	23 (3.0)	
Administrator/Managerial	33 (2.2)	22 (2.9)	11 (1.4)	
Data Capturer	23 (1.5)	9 (1.2)	14 (1.8)	
Other	257 (16.7)	122 (15.9)	135 (17.6)	

^a^ 11 missing age, 59 missing sex, 22 missing province.

^**b**^ Non-responders: 8 missing age, 50 missing sex, 19 missing province; responders: 3 missing age, 9 missing sex, 3 missing province.

^†^ Wilcoxen rank-sum test.

^††^ Chi-square tests for independence.

Of 1536 participants (median age = 36, 82.7% women), 350 (22.8%) responded to the six-month follow-up questionnaire. Of the 350 respondents, 283 (80.9%) were intervention group participants, and 67 (19.1%) were control group participants (Table 2). Those who received any intervention had 1.54 times higher odds of responding than controls (CI 1.15–2.07; *P* = 0.004). Type of messaging content was also associated with higher response (Table 2). Specifically, those that received themed messaging compared to control had increased odds of response (OR 1.68: CI 1.22–2.32; *P* = 0.001); no difference in response was found between generic messaging and controls, or between themed messaging and generic messaging. Choice of delivery method was associated with a higher response rate compared to control group (Group DM3; OR 1.60: CI 1.13–2.27; *P* = 0.008). However, no differences were found in other delivery methods compared to control, or between delivery methods. There was no interaction detected between delivery method and type of messaging content (*P* = 0.38, data not shown).

**Table 2:** Differences in response between combined interventions.

Type of Intervention	Specific Intervention	Response Outcome			
		Non-responder (n = 1186)	Responder (n = 350)	Odds ratio (95% CI)†	*P*-value††*
Control	Combined Control	317 (26.7)	67 (19.1)	Ref	-
All Interventions	**Messaging Content + Delivery Method**	**869 (73.3)**	**283 (80.9)**	**1.54 (1.15–2.07)**	0.004*
Messaging Content	**Themed Messaging (Group 1)**	**425 (35.8)**	**151 (43.1)**	**1.68 (1.22–2.32)**	0.001*
	Generic Messaging (Group 2)	444 (37.4)	132 (37.7)	1.41 (1.01–1.95)	0.041
Delivery Method	SMS Only (DM1)	294 (24.8)	90 (25.7)	1.45 (1.02–2.06)	0.040
	SMS + Postal (DM2)	288 (24.3)	96 (27.4)	1.58 (1.11–2.24)	0.010
	**Participant Choice (DM3)**	**287 (24.2)**	**97 (27.7)**	**1.60 (1.13–2.27)**	0.008*

^†^ OR and CI obtained at OpenEpi.com and confirmed with logistic regression.

^††^ Chi-square tests for independence.

*Bonferroni corrected significance for multiple tests (6), p < 0.00833. No differences found between Themed and Generic Messaging. No differences found between SMS Only and SMS + Postal; SMS Only and Participant Choice; or SMS + Postal and Participant Choice.

Differences in socio-demographic characteristics were found between non-responders and responders by age group, province and profession (Table 3). Compared to those less than 24 years old, odds of response was higher for participants aged 35–44 (OR 1.88: CI 1.22–2.88; *P* = 0.004). The odds of response for those participants from Gauteng Province were almost twice those in Limpopo Province (OR 1.94: CI 1.45–2.60; *P*<0.001). Compared to registered nurses, CHWs and allied health workers had 3.72 times (CI 2.63–5.26; *P*<0.001) and 4.53 times (CI 1.82–11.24; *P* = 0.004), respectively, greater odds of responding. Similarly, odds of response were also higher for lay counselors (OR 1.73: CI 1.07–2.80; *P* = 0.024) and educator/trainers (OR 2.07: CI 1.04–4.12; *P* = 0.036) compared to registered nurses. An assessment of the impact of health status on response rates revealed no differences between responders and non-responders by type or number of health conditions. (S1 Table).

**Table 3:** Differences in socio-demographic characteristics between responders and non-responders.

Socio-Demographics	Response Outcome			
	Non-Responders (n = 1186)	Responders (n = 350)	Odds ratio (95% confidence interval)†	*P*-value††
**Age Groups**				**0.039**
< 24	166 (14.1)	33 (9.5)	Ref	-
25–34	376 (31.9)	103 (29.7)	1.38 (0.89–2.12)	0.145
** 35–44**	**327 (27.8)**	**122 (35.2)**	**1.88 (1.22–2.88)**	**0.004**
45–54	238 (20.2)	71 (20.5)	1.50 (0.95–2.37)	0.08
> 55	71 (6.0)	18 (5.2)	1.28 (0.67–2.41)	0.45
Sex				0.83
Female	978 (82.5)	292 (83.4)	Ref	-
Male	158 (13.3)	49 (14.0)	1.04 (0.73–1.47)	0.83
**Province**				**<0.001**
Limpopo	384 (32.4)	79 (22.6)	Ref	-
Eastern Cape	15 (1.3)	2 (0.6)	0.65 (0.15–2.89)	0.86†††
Free State	40 (3.4)	4 (1.1)	0.49 (0.17–1.40)	0.24†††
** Gauteng**	**503 (42.4)**	**201 (57.4)**	**1.94 (1.45–2.60)**	**<0.001**
Kwazulu-Natal	0 (0.0)	1 (0.3)	-	0.35
Mpumalanga	179 (15.1)	52 (14.9)	1.41 (0.95–2.09)	0.084
Northern Cape	28 (2.4)	3 (0.9)	0.52 (0.15–1.76)	0.42†††
North West	6 (0.5)	0 (0.0)	-	0.66†††
Western Cape	12 (1.0)	5 (1.4)	2.03 (0.69–5.91)	0.32
**Profession**				**<0.001**
Registered Nurse	338 (28.5)	56 (16.0)	Ref	-
Enrolled Nurse	105 (8.9)	21 (6.0)	1.21 (0.70–2.09)	0.50
Auxiliary Nurse	68 (5.7)	11 (3.1)	0.98 (0.49–1.96)	0.95
** Lay Counselor**	**115 (9.7)**	**33 (9.4)**	**1.73 (1.07–2.80)**	**0.024**
** Community Health Worker**	**250 (21.1)**	**154 (44.0)**	**3.72 (2.63–5.26)**	**<0.001**
** Allied Health Worker**	**12 (1.0)**	**9 (2.6)**	**4.53 (1.82–11.24)**	**0.004**
** Educator/Trainer**	**38 (3.2)**	**13 (3.7)**	**2.07 (1.04–4.12)**	**0.036**
Administrator/Managerial	24 (2.0)	9 (2.6)	2.26 (1.00–5.12)	0.045
Data Capturer	20 (1.7)	3 (0.9)	0.91 (0.26–3.15)	>0.99†††
Other	216 (18.2)	41 (11.7)	1.15 (0.74–1.77)	0.54

^†^ OR and CI obtained at OpenEpi.com and confirmed with logistic regression.

^††^ Chi-square tests for independence.

^†††^ Fisher’s exact.

A difference in how surveys were returned (S2 Table) was found by type of delivery method, with odds of completing and returning the survey online 3.46 times higher for DM2 participants (CI 1.57–7.62; *P* = 0.001) compared to DM1. Interestingly, even if not used during interventions, participants who provided an email address (n = 495) at time of enrollment and responded to the follow-up survey (n = 100) had 7.57 greater odds of returning the survey online than those who did not provide an email address (CI 4.04–14.19; *P*<0.001) (S3 Table).

Enrollment logistics resulted in differences in the time to first SMS (2 to 248 days). Though no difference was found between responders and non-responders for time to first contact (S4 Table), other differences emerged for intervention participants who waited longer than three months to first contact. Among those, DM2 and DM3 participants had 2.07 (CI 1.29–3.33; *P* = 0.002) and 1.71 (CI 1.06–2.75; *P* = 0.03) higher odds of response, respectively, compared to DM0. Furthermore, participants from Gauteng had 2.28 higher odds of response compared to participants from Limpopo (CI 1.53–3.39; *P*<0.001), and CHWs responded better than registered nurses (OR 4.30; CI 2.37–7.81; *P*<0.001).

Of the 931 participants for whom contact tracing was attempted, 495 (53.2%) were reached, of whom 79 (16.0%) responded to the follow-up survey. Of these, 71 were from intervention groups (n = 283), increasing retention by 25.1%. Reasons cited by those reached and said they did not respond (n = 81; 16.4%) included: no time; not interested; sick; did not understand the questions; did not recall participating in the study (data not shown).

Differences in time to respond, using date of return, were found with themed messaging and DM2 (Table 4). After contact tracing, odds of response were 3.13 times greater for those participants who received themed messaging (CI 1.38–7.08; *P* = 0.005) compared to control. Similarly, DM2 participants, compared to control, had 3.35 times increased odds of returning the survey after contact tracing (CI 1.43–7.88; *P* = 0.004). An association between Madmaker response and survey response (S5 Table) was also found for participants who received themed messaging, with odds of response 4.89 times greater compared to those who received generic messaging (CI 1.20–19.94; *P* = 0.048).

**Table 4:** Differences in time to respond between interventions.

Type of Intervention	Specific Intervention	Response Outcome			
		Prior to Contact Tracing (n = 271)	After Contact Tracing (n = 79)	Odds ratio (95% CI)†	*P*-value††*
Control	Control	59 (21.8)	8 (10.1)	Ref	-
Messaging Content	**Themed Messaging**	**106 (39.1)**	**45 (57.0)**	**3.13 (1.38–7.08)**	**0.005**
	Generic Messaging	106 (39.1)	26 (32.9)	1.81 (0.77–4.25)	0.17
Delivery Method	SMS Only (DM1)	71 (26.2)	19 (24.1)	1.97 (0.81–4.83)	0.13
	**SMS + Postal (DM2)**	**66 (24.4)**	**30 (38.0)**	**3.35 (1.43–7.88)**	**0.004**
	Participant Choice (DM3)	75 (27.7)	22 (27.8)	2.16 (0.90–5.21)	0.08

^†^ OR and CI obtained at OpenEpi.com and confirmed with logistic regression.

^††^ Chi-square tests for independence.

*Bonferroni corrected significance for multiple tests (5), p < 0.01. No differences found between Themed and Generic Messaging. No differences found between SMS Only and SMS + Postal; SMS Only and Participant Choice; or SMS + Postal and Participant Choice.

Undeliverable mail (n = 93) was noted for survey delivery up to nine months after it was mailed. Of those, five (5.4%) participants responded to the online questionnaire. There was no discernable pattern of return timing.

## Discussion

Our study is the first known quantification of retention communication effectiveness in Africa aimed at developing retention strategies to reduce loss to follow-up. We found themed messages increased odds of response, a finding consistent with Booker *et al*. [12], which showed that themed messaging had a measureable effect on participant retention. Our quantitative findings also show that altruistic messaging improves retention, particularly among CHWs, allied health workers, lay counselors, and educator/trainers, and helps explain why these groups were more likely to respond no matter how long they waited for initial contact. This finding is supported Méjean *et al*. [18], who also found that altruistic messaging improves retention. This finding also supports the health belief model and importance of understanding appropriate benefits to appeal to specific audiences [9]. As shown in previous surveys with NIOH in South Africa, registered nurses were difficult to retain, possibly due to survey fatigue and staff shortages. This differs from nurses in the Harvard Nurses’ Health Study, which retained over 90% in the initial cohort [19]. Further sub-group analysis is needed to determine if themed messaging had a greater effect on response rate for one profession over another.

In our study, regular contact of any kind was associated with higher response rates, consistent with approaches and findings of previous studies [12, 13]. These studies illustrate the importance of having a systematic plan for participant contact that includes multiple points of contact and reminder messages to boost retention. Furthermore, participants who were given a choice between receiving either SMS or emails (DM3), and those that received both themed messaging and responded to a Madmaker had increased odds of response. This highlights the importance and association between participant engagement with the study team and increased odds of response. Early engagement with participants in two-way communication, via technology like Madmaker and SMS, especially in mobile populations like South Africa, should be expanded.

Participants who received more detailed information through both SMS and postal communication interventions (DM2) had increased odds of responding online, demonstrating the use of one medium to encourage engagement of another. Higher odds of response after contact tracing from participants who received themed messaging and DM2 stresses the importance of adding the personal link between participants and study staff. This supports previous study findings and multichannel commercial marketing best practices that using multiple contact strategies (including contact tracing) improved retention [13] [14]. Researchers should consider having multiple response options regardless of the contact method and offer more detailed study information delivered through multiple modes of communication, including reminder messages.

We found that odds of response for participants aged 35–44 was better than those aged 24 and younger. Although one study [12] found that younger participants were more likely to respond after reminder messaging, evidence from other studies is inconsistent with respect to the age of participants [6]. However, employment, marriage, higher education and socioeconomic status are associated with higher study participation. In South Africa, high unemployment and mobility among younger participants could contribute to higher instability and partly explain their lower response rates in our study. More effort and different messaging and delivery methods might be required to engage with younger audiences. Similarly, unique characteristics of rural participants in provinces like Limpopo might have affected retention, suggesting a different mix of messaging and delivery methods may be needed to reach and effectively retain those in rural communities compared to more urban communities.

Given that messaging was not initiated until our sample size was reached, which took six months, there was great variability in the time-from-enrollment to first contact between participants. Although we expected this to be a limitation based on best practices from the Harvard Nurses’ Health Study [19], which makes immediate contact with newly enrolled participants, no difference was found between those who waited more than or less than three months before initial contact. This suggests that there may be flexibility in the length of time before initial contact is made. Researchers should consider possible greater economies of scale when making initial contact with study participants by grouping participants into larger bulk messaging formats. This could potentially save scarce resources and time with no measurable impact on retention.

Despite statistics showing relatively high access to the Internet, most South Africans can only access it through their phone, which may limit or inhibit web surfing [10]. In our study, presence of an email address was strongly associated with online response, suggesting greater influence on response than simply having Internet access on a phone, which can make completing long surveys cumbersome or potentially costly without data bundles and contracts. Even with multiple SIM cards and changing phone numbers, wide use and acceptance of SMS enable a practical and cost-effective way to maintain cohort participation in South Africa. Finally, though small, the monetary incentive offered during the original questionnaire may have contributed to less than fully committed participation during follow up. Future studies may consider incentives during follow up instead of during enrollment.

Challenges abound for maintaining contact with cohort participants in South Africa from changing mobile phone numbers to unreliable postal delivery. Invalid or illegible contact details, or use of multiple SIM cards may have resulted in participants never having received any of the planned interventions. Although we attempted to address this concern in the study introduction script and by asking for updated contact details throughout the study, developing additional procedures to verify contact information before a participant completes their enrollment and throughout the study will help decrease the impact of invalid or illegible contact details. During 2014–2015, postal strikes likely impacted timing and delivery as well as survey returns. Because difficulties in delivering mail to townships contributed to undeliverable mail being returned, future studies should explore the use of more cost-effective, innovative mobile technologies to improve survey delivery and response.

A higher retention rate is required to ensure the validity of a cohort study. By identifying factors associated with responders and non-responders, our study supports the importance of themed messages and regular contact in retention strategies, and will inform the tailoring of initial enrollment of future participants to increase subsequent retention rates.

## Supporting information

S1 Appendix Follow-up questionnaire sample. (PDF)

S1 Table Differences in health status between responders and non-responders. (DOCX)

S2 Table Differences in method of survey return between interventions. (DOCX)

S3 Table Differences in response by email provided and method of survey return. (DOCX)

S4 Table Differences in length of time to first contact between responders and non-responders. (DOCX)

S5 Table Differences in response within interventions associated with response to first Madmaker. (DOCX)
