# Supplementary material for: The South African Rea Phela Health Study: A randomized controlled trial of communication retention strategies
Source: PLoS One. 2018 May 24;13(5):e0196900. doi: 10.1371/journal.pone.0196900 (PMC5967788; doi:10.1371/journal.pone.0196900)
Supplement: S1 Appendix — (PDF) [file pone.0196900.s001.pdf]

Rea Phela
HEALTH STUDY

Study number 10001

INSTRUCTIONS

1. Fill in only ONE box for each question, unless otherwise asked for.
2. Use a soft PENCIL only.
3. Darken the circle completely.
4. Fill in the blocks at the top as seen in the example to the right.

CORRECT MARK

INCORRECT MARKS

Example:

2 8 0 7 2 0 1 3

FOR OFFICIAL USE ONLY

Study Number

1. Today's Date

D D M M 2 0 Y Y

2. Date of Birth

D D M M Y Y Y Y

3. Cellphone Number

4. Age

5. Have you relocated in the past 6 months?

- Yes
- No

6. Have you changed your place of work in the past 6 months?

- Yes
- No

7. What is your profession?

- Medical Doctor
- Registered Nurse
- Enrolled Nurse
- Auxiliary Nurse
- Lay Counsellor
- Community Health Care Worker
- Allied Health Care Worker
- Educator / Trainer
- Administrator / Managerial
- Data capturer
- Other, specify: ____________

8. Have you earned a new qualification in the past 6 months? For example, your diploma or a Master's degree. Do not include certificates of attendance or participation

- Yes
- No

9. In the past 6 months, what was your dominant / primary function at work? (Choose one)

- Clinical: direct patient care and treatment
- Clinical support: pharmacy, counselling, facility-based social work
- Management
- Administration
- Research Consulting,Technical Advising and Training
- Education / Training
- Community-based outreach and support
- Other

10. How much money comes into your household each month? (After tax deductions. Include all sources)

- None
- Less than R1000
- R1001 - R2000
- R2001 - R4000
- R4001 - R6000
- R6001 - R8000
- R8001 - R10 000
- R10 001 - R15 000
- R15 001 - R20 000
- More than R20 000

11. How many people use this money for basic needs like food, rent, school fees, medical care, or transport, including yourself?

- 1 to 2
- 3 to 5
- 6 to 7
- 8 to 9
- 10 or more

12. Are you currently employed (working for pay)?	
Yes	○
No	○

If No, skip to question 17

Questions 13 to 17 are about the past seven days, not including today.

13. During the past seven days, how many hours did you miss from work because of your health problems? *Include hours you missed on sick days, times you went in late, left early, etc., because of your health problems.*

None	○	5 to 8 hours	○	17 to 24 hours	○
1 to 4 hours	○	9 to 16 hours	○	More than 24 hours	○

14. During the past seven days, how many hours did you miss from work because of any other reason, such as vacation or holidays?

None	○	5 to 8 hours	○	More than 24 hours	○
1 to 4 hours	○	9 to 16 hours	○		

15. During the past seven days, how many hours did you actually work?

Less than 20 hours	○	40 hours	○	More than 60 hours	○
20 to 39 hours	○	41 to 60 hours	○		

16. During the past seven days, how much did your health problems affect your productivity while you were working?

If health problems affected your work only a little, choose a low number. Choose a high number if health problems affected your work a great deal.

Consider only how much health problems affected productivity while you were working

Health problems had no effect on my work	0	1	2	3	4	5	6	7	8	9	Health problems completely prevented me from working

17. During the past seven days, how much did your health problems affect your ability to do your regular daily activities, other than work at a job?

If health problems affected your activities only a little, choose a low number. Choose a high number if health problems affected your activities a great deal.

Consider only how much health problems affected your ability to do your regular daily activities, other than work at a job

Health problems had no effect on my daily activities	0	1	2	3	4	5	6	7	8	9	Health problems completely prevented me from doing my daily activities

18. In the last 6 months, have you been diagnosed by a clinician with any of the following? (Mark all that apply)

Breast cancer	○	Asthma	○
Cervical cancer	○	Tuberculosis	○
Colorectal cancer	○	HIV	○
Liver cancer	○	Arthritis	○
Other type of cancer not listed	○	General chronic joint pain	○
Diabetes	○	Lower back pain	○
Hyperthyroidism or hypothyroidism	○	Chronic headaches	○
Elevated cholesterol	○	Epilepsy	○
Hypertension / High blood pressure	○	Depression	○
Myocardial infarction (heart attack)	○	Anxiety	○
Congestive heart failure	○	Ulcer	○
Stroke	○	Kidney stones	○
Blood clots	○	Gum disease	○

19. Mark all medications that you have used at least 2 times per week over the past 2 years			
Paracetamol (Panado)	○	COX-2 inhibitors (Coxflam)	○
Aspirin or aspirin-containing products	○	Codeine-compound painkillers	○
Ibuprofen (Brufen)	○	Other anti-inflammatory analgesics (Voltaren, Diclofenac)	○
Thiazide diuretic (Hydrochlorothiazide)	○		
Furosemide (Lasix, Puresis)	○	Opioid pain medications	○
Potassium (Slow K)	○	Evista (Raloxifen)	○
Beta-blocker (Tembloka)	○	Nolvadex (Tamoxifen)	○
ACE inhibitors (Pharmapress, Prexum)	○	SSRIs (Cipralex, Prozac)	○
Angiotensin receptor blocker (Losartan or Micardis)	○	Tricyclics (Trepeline)	○
Warfarin	○	SNRIs / Other antidepressants (Effexor, Cymbalta)	○
Pradaxa / Xarelto / Eliquis	○	Minor tranquilisers (Valium)	○
Plavix	○	Antiacids (Rennies)	○
Statin cholesterol-lowering drug (Simvotin, Crestor)	○	Bisphophonate (Fosamax for osteoporosis)	○
Non-statin cholesterol-lowering drug	○	Thyroid hormone (Eltroxin)	○
Steroids taken orally	○	Prescription sleeping tablets (Stilnox)	○
Insulin	○	Cough medicine containing codeine (Benylin with Codeine)	○
Metformin (Glucophage)	○		
Other oral diabetes medication (Amaryl, Diamicronet)	○	Anti-retroviral medications	○
		Other regular medications	○

20. When did you last take antibiotics?			
In the past week	○	Over one year ago	○
In the past month	○	I have never taken antibiotics	○
In the past 6 months	○	I do not know / I do not remember	○
In the last year	○		

21. Over the <u>last two weeks</u> how often have you been bothered by any of the following problems?	Not at all	Several Days	More than half the days	Nearly every day
a) Little interest or pleasure in doing things	○	○	○	○
b) Feeling down, depressed or hopeless	○	○	○	○
c) Trouble falling or staying asleep, or sleeping too much	○	○	○	○
d) Feeling tired or having little energy	○	○	○	○
e) Poor appetite or overeating	○	○	○	○
f) Feeling bad about yourself - or that you are a failure or have let yourself or your family down	○	○	○	○
g) Trouble concentrating on things, such as reading the newspaper or watching television	○	○	○	○
h) Moving or speaking so slowly that other people have noticed. Or the opposite - being so fidgety or restless that you have been moving around a lot more than usual	○	○	○	○
i) Thoughts that you would be better off dead, or of hurting yourself	○	○	○	○
j) If you checked <u>any</u> of these problems, how <u>difficult</u> have these problems made it for you to do your work, take care of things at home, or get along with other people?	Not difficult at all ○		Very difficult ○	
	Somewhat difficult ○		Extremely difficult ○	

22. Over the <u>last two weeks</u> how often have you been bothered by any of the following problems?	Not at all	Several Days	More than half the days	Nearly every day
a) Feeling nervous, anxious or on edge	○	○	○	○
b) Not being able to stop or control worrying	○	○	○	○
c) Worrying too much about different things	○	○	○	○
d) Trouble relaxing	○	○	○	○
e) Being so restless that it is hard to sit still	○	○	○	○
f) Becoming easily annoyed or irritable	○	○	○	○
g) Feeling afraid as if something awful might happen	○	○	○	○
h) If you checked <u>any</u> of these problems, how <u>difficult</u> have these problems made it for you to do your work, take care of things at home, or get along with other people?	Not difficult at all ○		Very difficult ○	
	Somewhat difficult ○		Extremely difficult ○	

**23. The following questions are about very stressful or traumatic events that might have happened in your life.**	**Never**	**1 - 5 years ago**	**6 - 10 years ago**	**11 - 20 years ago**	**More than 20 years ago**
a) Have you ever been involved in a life-threatening automobile accident?	◯	◯	◯	◯	◯
b) Have you ever had any other life-threatening accident, including on your job?	◯	◯	◯	◯	◯
c) Have you ever been mugged, held up, or threatened with a weapon?	◯	◯	◯	◯	◯
d) The next question is about rape. We define rape as an event during which one person has sexual intercourse with or penetrates the body of another person **without** their consent, or when they were too young to know what was happening. Did you ever have an experience like this?	◯	◯	◯	◯	◯
e) The next question is about sexual assault. We define sexual assault as an event during which one person touches another person inappropriately, or **without** that person's consent. Did you ever have an experience like this?	◯	◯	◯	◯	◯
f) Has someone very close to you ever died unexpectedly? (For example, they were killed in an accident, murdered, committed suicide, or died of a disease at a young age)	◯	◯	◯	◯	◯
g) Have you ever had a son or daughter who had a life-threatening illness or injury?	◯	◯	◯	◯	◯
h) Have you ever seen someone being badly injured or killed, or unexpectedly see a dead body?	◯	◯	◯	◯	◯
i) Have you ever had any other extremely traumatic or life-threatening event?	◯	◯	◯	◯	◯

If you answered "Never" to all items on question 23 (a through i), skip to question 25.

**24. Please now think about the one traumatic event that you experienced that caused you the most problems. Thinking of this particular traumatic event, how often have you experienced the following since the event?**	**Rarely**	**Some-times**	**Most of the time**	**All of the time**
a) Recurrent thoughts or memories of the event	◯	◯	◯	◯
b) Feeling as though the event is happening again	◯	◯	◯	◯
c) Recurrent nightmares about the event	◯	◯	◯	◯
d) Sudden emotional or physical reactions when reminded of the event	◯	◯	◯	◯
e) Avoiding activities that remind you of the event	◯	◯	◯	◯
f) Avoiding thoughts or feelings associated with the event	◯	◯	◯	◯
g) Feeling jumpy, easily startled	◯	◯	◯	◯
h) Feeling on guard	◯	◯	◯	◯

**25. How often do you exercise?**	
6 or more times per week	◯
3 to 5 times per week	◯
1 to 2 times per week	◯
A few times per month	◯
Less than one time per month	◯
Never	◯

If Never, skip to question 28

**26. When you do exercise, how long do you exercise for?**	
More than 30 minutes	◯
20 to 30 minutes	◯
10 to 20 minutes	◯
Less than 10 minutes	◯

27. When you exercise, how hard do you exercise? (Choose one)		
High intensity activities that result in sustained heavy breathing and perspiration (fast walking, high impact aerobics, running fast, swimming, playing soccer, cycling in competition, netball match)	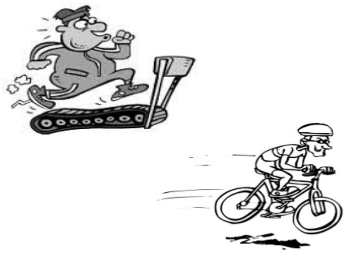	○
Moderately high aerobic activities or intermittent sports activities that result in sustained heavy breathing and perspiration (step aerobics, stair stepping, speed walking, tennis, racquet ball, squash)	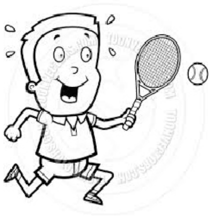	○
Moderate aerobic activities (normal bike riding, jogging, low impact aerobics)	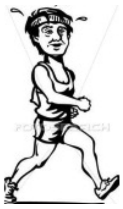	○
Low to moderate aerobic and sports activities (recreational volleyball, moderate speed walking, fetching water or wood, dancing, working in the garden, cleaning windows, golfing, walking with your dog, walking to the taxi)	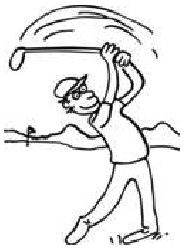	○
Light aerobic exercise (normal walking, light household duties)	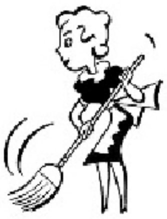	○

28. On average, how many hours per day do you spend doing any of the following: sitting in meetings, sitting in front of your computer or cell phone, watching television or reading?

⓪	①	②	③	④	⑤	⑥	⑦	⑧	⑨	10+

EXAMPLES:

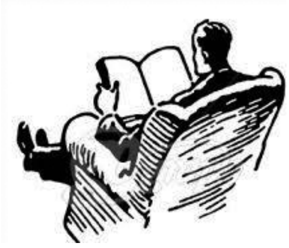

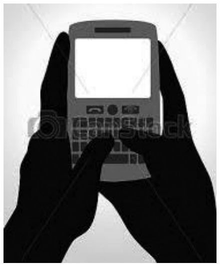

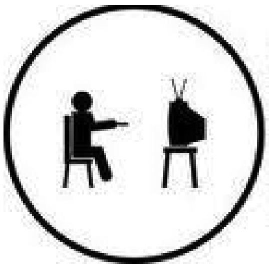

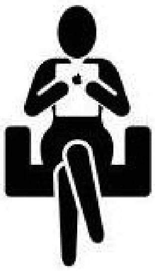

29. How much do you weigh? (In kilograms)

0	0	0
1	1	1
2	2	2
3	3	3
4	4	4
5	5	5
6	6	6
7	7	7
8	8	8
9	9	9

30. How tall are you? (In meters)

,		
0	0	0
1	1	1
2	2	2
3	3	3
4	4	4
5	5	5
6	6	6
7	7	7
8	8	8
9	9	9

**31. When was the last time you saw a dentist?**	
In the past 6 months	◯
More than 6 months ago, but not more than 1 year ago	◯
More than 1 year ago, but not more than 2 years ago	◯
More than 2 years ago, but not more than 3 years ago	◯
More than 3 years ago, but not more than 5 years ago	◯
More than 5 years ago	◯
I have never been to a dentist	◯

**32. What was the main reason of your last visit to the dentist?**	
Check-up, examination, or teeth cleaning	◯
Symptoms (Something was wrong, bothering, or hurting you)	◯
Went for treatment of a condition found at a previous examination	◯

**33. How frequently do you do the following to take care of your teeth?**	**At least twice a day**	**Once a day**	**5 - 6 times a week**	**3 - 4 times a week**	**1 -2 times a week**	**Almost never**
a) Brush your teeth	◯	◯	◯	◯	◯	◯
b) Floss your teeth	◯	◯	◯	◯	◯	◯
c) Rinse with mouth wash (e.g. Listerine)	◯	◯	◯	◯	◯	◯

**34. As an adult, have you ever:**	
Had a tooth extracted	◯
Lost a tooth that was not replaced	◯
Both of the above	◯
Neither	◯

**35. Have you had any of the following oral health conditions?**	**Within the last year**	**More than 1 year ago**	**Never**
a) Toothache	◯	◯	◯
b) Sore or sensitive tongue	◯	◯	◯
c) Teeth sensitive to cold or heat	◯	◯	◯
d) Chronic bad breath	◯	◯	◯
e) Bleeding gums	◯	◯	◯
f) Ulcers / sores in the mouth	◯	◯	◯
g) Loose teeth	◯	◯	◯

36. Over the past 3 months:

a) How many times a week did you usually eat fast food meals or snacks?

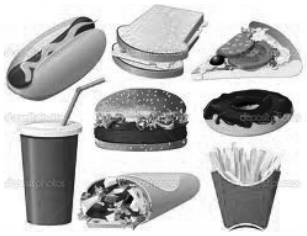

- Less than 1 time ◯
- 1 – 3 times ◯
- 4 or more times ◯

b) How many regular (exclude “diet” drinks like Coke Lite) cool drinks or glasses / cups of sweet tea or coffee did you drink each day?

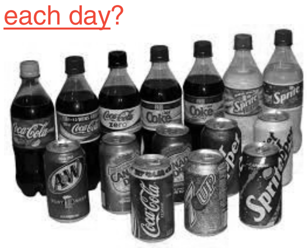

- Less than 1 ◯
- 1 - 2 ◯
- 3 or more ◯

c) How many servings of fruit did you usually eat each day?

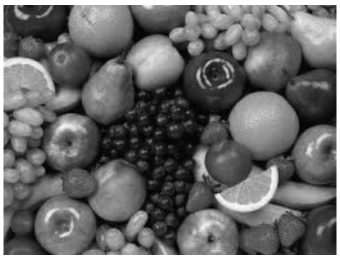

- 5 or more ◯
- 3 – 4 ◯
- 2 or less ◯

d) How many servings of vegetables did you usually eat each day?

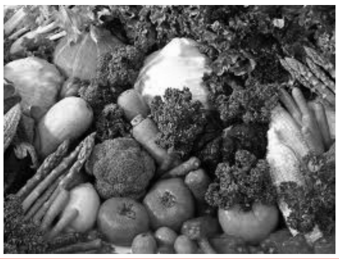

- 5 or more ◯
- 3 – 4 ◯
- 2 or less ◯

e) How many times a week did you eat legumes (dried beans like sugar beans, peas, lentils), chicken or fish?

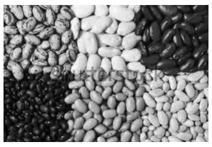

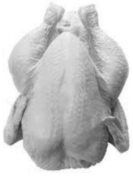

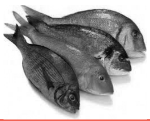

- 3 or more times ◯
- 1 - 2 times ◯
- Less than 1 time ◯

f) How many times a week did you eat crisps (e.g. Simba) or salty crackers (not low-fat)?

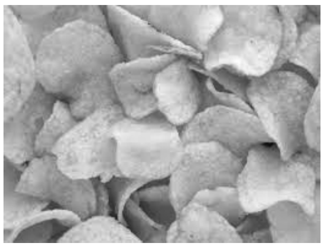

- 1 time or less ◯
- 2 - 3 times ◯
- 4 or more times ◯

g) How many times a week did you eat desserts / cookies / biscuits / cake (not low-fat)?

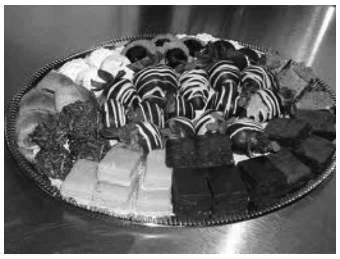

- 1 time or less ◯
- 2 - 3 times ◯
- 4 or more times ◯

h) How much margarine, butter, mayonnaise, oil or meat fat (sauce) did you use with food (vegetables, potatoes, bread, porridge, etc.)?

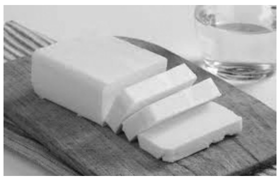

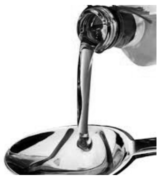

- Very little ◯
- Some ◯
- A lot ◯

i) How many times a week did you usually eat red meat (beef, mutton, pork, sausages)?

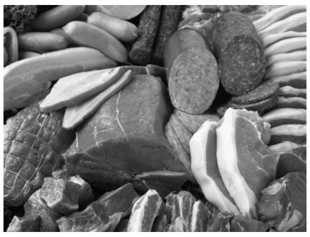

- 6 or more times ◯
- 3 - 5 times ◯
- 2 times or less ◯

j) How many servings of dairy (milk, maas, yoghurt, cheese) did you usually have each day?

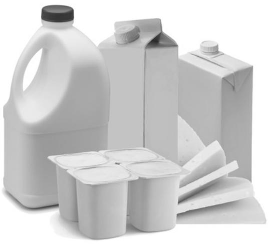

- Less than 1 ◯
- 1 - 2 ◯
- 3 or more ◯

37. Consider the statement: "I feel safe in my neighbourhood." Do you:

Strongly agree	◯	Disagree	◯
Agree	◯	Strongly disagree	◯

38. Do you think the following items are a problem in your neighbourhood?	Big problem	Somewhat of a problem	Not a problem
a) Vacant areas with trash or junk	○	○	○
b) Litter, broken glass, or trash on the sidewalks and streets	○	○	○
c) People fighting or arguing	○	○	○
d) Vacant or deserted housing or storefronts	○	○	○
e) Vandalism, like people breaking windows or wind screens	○	○	○
f) Inadequate police protection	○	○	○
g) Loud noises that wake you up or keep you awake	○	○	○
h) People selling or using drugs	○	○	○
i) Groups of teenagers or adults hanging out and causing trouble	○	○	○
j) Graffiti on buildings and walls	○	○	○
k) Drinking alcohol in public	○	○	○
l) Assaults, muggings, or burglaries	○	○	○

The next questions are about things that happen to many people, and that your current partner, or any other partner may have done to you. Colour in the appropriate circle(s).

39. Has your current spouse / partner or any other partner ever:	Has this happened to you in the past 12 months? No	Yes	If yes, in the past 12 months, how many times would you say that this has happened?
a) Insulted you or made you feel bad about yourself?	Ⓝ	Ⓨ	One ○<br>Few ○<br>Many ○
b) Belittled you or humiliated you in front of other people?	Ⓝ	Ⓨ	One ○<br>Few ○<br>Many ○
c) Done things to scare or intimidate you on purpose (for example, by the way he / she looked at you, by yelling and smashing things)?	Ⓝ	Ⓨ	One ○<br>Few ○<br>Many ○
d) Verbally threatened to hurt you or someone you care about?	Ⓝ	Ⓨ	One ○<br>Few ○<br>Many ○
e) Slapped you or thrown something at you that could hurt you?	Ⓝ	Ⓨ	One ○<br>Few ○<br>Many ○
f) Pushed or shoved you or pulled your hair?	Ⓝ	Ⓨ	One ○<br>Few ○<br>Many ○
g) Hit you with his / her fist or with something else that could hurt you?	Ⓝ	Ⓨ	One ○<br>Few ○<br>Many ○
h) Kicked you, dragged you or beaten you up?	Ⓝ	Ⓨ	One ○<br>Few ○<br>Many ○
i) Choked or burnt you on purpose?	Ⓝ	Ⓨ	One ○<br>Few ○<br>Many ○
j) Threatened with or used a gun, knife or other weapon against you?	Ⓝ	Ⓨ	One ○<br>Few ○<br>Many ○
k) Forced you to have sexual intercourse when you did not want to, for example by threatening you or holding you down?	Ⓝ	Ⓨ	One ○<br>Few ○<br>Many ○
l) Did you ever have sexual intercourse when you did not want to because you were afraid of what your spouse / partner might do if you refused?	Ⓝ	Ⓨ	One ○<br>Few ○<br>Many ○
m) Did your spouse / partner ever force you to do anything else sexual that you did not want or that you found degrading and humiliating?	Ⓝ	Ⓨ	One ○<br>Few ○<br>Many ○

40. Are you afraid of your current / most recent spouse / partner?

Never ○	Often ○
Sometimes ○	Most of the time ○

41. In the past 12 months, have you ever slapped, pushed, hit or physically mistreated your spouse / partner when he / she was not slapping, pushing, hitting or physically mistreating you?

Never ○	2 - 5 times ○
Once ○	More than 5 times ○

The following questions are for women only. If you are a man, please skip to question 49

42. Please read the following statements and think about your relationship with your current husband or boyfriend. For each statement mark if you strongly agree, agree, disagree or strongly disagree	Strongly agree	Agree	Disagree	Strongly disagree
a) If I ask my partner to use a condom, he would beat or hit me	○	○	○	○
b) If I ask my partner to use a condom, he would get angry	○	○	○	○
c) My partner won't let me wear certain things	○	○	○	○
d) My partner has more to say than I do about important decisions that affect us	○	○	○	○
e) My partner tells me who I can spend time with	○	○	○	○
f) I could leave our relationship any time I want to	○	○	○	○
g) My partner does what he wants, even if I don't want him to	○	○	○	○
h) If I ask my partner to use a condom, he would think that I'm having sex with other people	○	○	○	○
i) When my partner and I disagree, he gets his way most of the time	○	○	○	○
j) Because my partner buys me things, he expects me to please him	○	○	○	○
k) My partner always wants to know where I am	○	○	○	○

43. Have you been pregnant or given birth during the past 6 months?

Yes ○
No ○

44. For any pregnancy or birth in the past 6 months, did you experience any of the following complications: (Mark all that apply)

Gestational diabetes ○	Pre-term labour ○	Full term, low birth weight ○
Placenta previa ○	Infection ○	None ○
Post-partum haemorrhage ○	Anaemia ○	Other ○
Post-partum depression ○	Pre-eclampsia ○	I have not been pregnant or given birth during the past 6 months ○
Spontaneous abortion ○	Still birth ○	

45. In the past 6 months, have you used any of the listed Hormonal Contraceptives? (Mark all that apply)

Oral contraception (Femodene, Biphasil, Ovral, Nordette) ○	I have used hormonal contraception in the last 6 months, but I do not remember which type ○
Injectable contraception (Depo Provera, Nur-Isterate) ○	I have not used hormonal contraception in the last 6 months ○
Intrauterine device (Mirena, SMB Corp) ○	
Combined hormonal patch (Evra) ○	
Combined hormonal vaginal ring ○	
Subdermal implant (Norplant, Sino-Plant, Jadelle) ○	

46. Have you ever taken hormone replacement therapy (HRT) or estrogen therapy?

Yes, I am currently on HRT or estrogen therapy ○
Yes, I was on HRT or estrogen therapy in the past, but I am not using it currently ○
No, I have never used HRT or estrogen therapy ○

47. At what age did you start HRTor estrogen therapy?

Less than 45 years old ○	Older than 56 years ○
45 - 50 years old ○	I have never used HRT or estrogen therapy ○
51 - 56 years old ○	

48. For how long have you been or were you on HRT or estrogen therapy?

Less than 1 year ○	7 - 9 years ○
1 - 3 years ○	10 or more years ○
4 - 6 years ○	I have never used HRT or estrogen therapy ○

49. In the past 6 months, have you been diagnosed by a clinician with any of the following: (Mark all that apply)

Vaginal infection ○	Syphilis ○	Genital herpes ○
Pelvic Inflammatory Disease (PID) ○	Chlamydia ○	Other ○
Urinary Tract Infection (UTI) ○	Gonorrhoea ○	None ○
Human Papillomavirus (HPV) ○	Trichomoniasis ○	

50. Where do you typically go for medical care? (Choose one)			
Public hospital	◯	Private hospital	◯
Public clinic	◯	Private doctor	◯

51. When did you last visit a traditional healer?			
In the past year	◯	Never	◯
More than a year ago	◯		

52. When was the last time you went for one of these tests?	Within the last year	1 to 3 years ago	4 to 5 years ago	More than 5 years ago	Never
a) Blood pressure or hypertension	◯	◯	◯	◯	◯
b) Fasting or non-fasting glucose level	◯	◯	◯	◯	◯
c) Cholesterol	◯	◯	◯	◯	◯
d) Prostate exam (Men only)	◯	◯	◯	◯	◯
e) PSA test (Men only)	◯	◯	◯	◯	◯
f) Pap smear (Women only)	◯	◯	◯	◯	◯
g) Clinical breast exam (Women only)	◯	◯	◯	◯	◯

h) Self-breast exam in the last month	Yes	No
	◯	◯

53. When were you last tested for HIV?	
Within the last year	◯
1 to 3 years ago	◯
4 to 5 years ago	◯
More than 5 years ago	◯
Never	◯

54. What was the result of your most recent test?	
Negative	◯
Positive	◯
I do not know	◯
It is a private matter / I do not want to answer this question	◯

55. If positive, are you taking HIV medication?	
Yes	◯
No	◯
It is a private matter / I do not want to answer this question	◯

56. Have you ever smoked at least 100 cigarettes (5 packs of cigarettes) or the equivalent amount of tobacco in your lifetime (this excludes snuff and hubbly bubbly)?		
Yes	◯	
No	◯	If no, skip to question 62

57. When did you start smoking cigarettes?	
Less than 1 year ago	◯
1 - 5 years ago	◯
6 - 10 years ago	◯
11 - 15 years ago	◯
More than 15 years ago	◯

58. How long has it been since you last smoked a cigarette, even one or two puffs?	
Within the past month	◯
1 - 3 months ago	◯
4 - 6 months ago	◯
7 - 12 months ago	◯
1 - 5 years ago	◯
6 - 10 years ago	◯
More than 10 years ago	◯

59. During the past 30 days how many days did you smoke cigarettes or tobacco products?	
0 days	◯
1 or 2 days	◯
3 to 5 days	◯
6 to 9 days	◯
10 to 19 days	◯
20 to 29 days	◯
All 30 days	◯

Please answer this question even if you did not smoke cigarettes in the last 30 days.

60. On the days that you smoked, how many cigarettes did you usually smoke each day?	
5 or less cigarettes per day	◯
6 to 10 cigarettes per day	◯
11 to 20 cigarettes per day	◯
More than 20 cigarettes per day	◯

61. How soon after you wake up do you usually smoke a cigarette for the first time?	
Within 5 minutes	◯
6 - 30 minutes	◯
31 - 60 minutes	◯
More than 60 minutes	◯
I do not currently smoke cigarettes	◯

62. Which type of snuff did / do you use?

- Oral ○
- Nasal ○
- Both ○
- I have never used snuff ○

63. During the past 30 days how many days did you use snuff?

- 0 days ○
- 1 or 2 days ○
- 3 to 5 days ○
- 6 to 9 days ○
- 10 to 19 days ○
- 20 to 29 days ○
- All 30 days ○
- I have never used snuff ○

Please answer this question even if you did not use snuff in the last 30 days.

64. On the days that you used snuff, how many times a day did you use snuff?

- 1 time per day ○
- 2 - 5 times per day ○
- 6 - 10 times per day ○
- 11 - 20 times per day ○
- More than 20 times per day ○
- I have never used snuff ○

65. In the past year, how often have you smoked hubbly bubbly?

- Never ○
- Monthly or less ○
- 2 - 3 times per month ○
- Once per week ○
- More than once per week ○

The next set of questions are about your use of alcoholic drinks during this past year.

For questions 66 - 68, one drink is equivalent to:

One 330ml bottle or can of beer

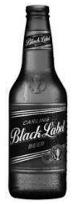

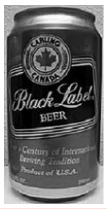

One 120ml glass of wine

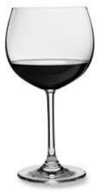

One shot or tot of alcohol

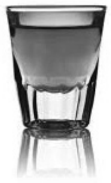

66. How often do you have a drink containing alcohol?

- Never ○
- Monthly or less ○
- 2 - 4 times per month ○
- 2 - 3 times a week ○
- 4 or more times a week ○

67. On a typical day when you are drinking, how many drinks containing alcohol do you drink?

- 1 or 2 ○
- 3 or 4 ○
- 5 or 6 ○
- 7, 8 or 9 ○
- 10 or more ○
- I have not had a drink containing alcohol in the past year ○

68. How often do you have six or more drinks on one occasion?

- Never ○
- Less than monthly ○
- Monthly ○
- Weekly ○
- Daily or almost daily ○
- I have not had a drink containing alcohol in the past year ○

69. Have you ever used chemical hair straighteners?

- Yes, I am currently using chemical hair straighteners ○
- Yes, I have used chemical hair straighteners in the past, but I am not using it currently ○
- No, I have never used chemical hair straighteners ○

70. At what age did you first use chemical hair straighteners?

- Less than 10 years old ○
- 10 - 19 years old ○
- 20 - 29 years old ○
- 30 years or older ○
- I have never used chemical hair straighteners ○

71. How many times per year do you use chemical hair straighteners?

- 1 time ○
- 2 - 4 times ○
- 5 - 6 times ○
- 7 - 8 times ○
- 9 - 12 times ○
- More than 12 times per year ○
- I have never used chemical hair straighteners ○

72. How many times have you had burns (a break in the skin, not just tingling) during the application of chemical hair straighteners?

- None ○
- Once or twice ○
- 3 - 4 times ○
- 5 - 9 times ○
- 10 or more times ○
- I have never used chemical hair straighteners ○

73. Have you ever used skin-lightening cream?
- Yes, I am currently using skin-lightening creams ○
- Yes, I have used skin-lightening creams in the past, but I am not using it currently ○
- No, I have never used skin-lightening creams ○

74. At what age did you first use skin-lightening creams?
- Less than 10 years old ○
- 10 - 19 years old ○
- 20 - 29 years old ○
- 30 years or older ○
- I have never used skin-lightening creams ○

75. How often do you or did you apply skin-lightening creams?
- Daily or almost daily ○
- 2 - 5 days per week ○
- Weekly ○
- A few times per month ○
- Monthly ○
- I have never used skin-lightening creams ○

76. On average, how much sleep do you get on a typical night when you are working the next day?
- Less than 5 hours ○
- 5 - 6 hours ○
- 7 - 8 hours ○
- More than 8 hours ○

77. On average, how much sleep do you get on a typical night when you are not working the next day?
- Less than 5 hours ○
- 5 - 6 hours ○
- 7 - 8 hours ○
- More than 8 hours ○

The following questions are about your experiences as a participant in the Rea Phela Health Study

78. Was the questionnaire:
- Too long ○
- The right length ○
- I would answer more questions ○

79. Overall, I had no problems or concerns taking this questionnaire
- Strongly agree ○
- Agree ○
- Disagree ○
- Strongly disagree ○

80. Overall, I thought the questions were confusing
- Strongly agree ○
- Agree ○
- Disagree ○
- Strongly disagree ○

81. Overall, I thought the answer sheet was confusing
- Strongly agree ○
- Agree ○
- Disagree ○
- Strongly disagree ○

82. Would you be willing to participate in a long-term study of health care workers in which you completed a questionnaire like this once every two years?
- Yes ○
- No ○

83. May we contact you in a few months to answer similar questions?
- Yes ○
- No ○

84. Why did you decide to complete the questionnaire you received during an FPD training? (choose one)
- Chance to win a Spar gift voucher ○
- Colleagues were doing it ○
- Personal or professional interest ○
- Easy to do - attending an FPD class ○
- Better health for all South Africans ○

85. Whether you were contacted by SMS, email or postal letter, how would you have preferred to be contacted?
- a) SMS ○
- b) Email ○
- c) Postal letter ○
- d) No contact ○

86. How did you feel about the number of times we contacted you about the Rea Phela Health Study? Did we contact you:
- Too few times ○
- Too many times ○
- The right amount ○

87. Did you "Like" the Facebook page for the Rea Phela Health Study?
- Yes ○
- No ○
- I did not know about the Facebook page ○

88. Did you visit the Rea Phela Health Study website at least once?
- Yes ○
- No ○
- I did not know about the study website ○

89. Did the information and messaging you received during the Rea Phela Health Study make you feel:
- No connection to the study ○
- A bit connected to the study ○
- Very connected to the study ○

THIS IS THE END OF THE QUESTIONNAIRE. THANK YOU FOR PARTICIPATING!
