# Supplementary material for: The South African Rea Phela Health Study: A randomized controlled trial of communication retention strategies
Source: PLoS One. 2018 May 24;13(5):e0196900. doi: 10.1371/journal.pone.0196900 (PMC5967788; doi:10.1371/journal.pone.0196900)
Supplement: S1 Table — (DOCX) [file pone.0196900.s002.docx]

|  | Intent-to-Treat | Response Outcome | |  | |
| --- | --- | --- | --- | --- | --- |
| Health Status | Participants (n=1536) | Non-Responders  (n=1186) | Responders (n=350) | Odds ratio (95% confidence interval)† | *P*-value†† |
| No Conditions | 982 (63.9) | 758 (63.9) | 224 (64.0) | Ref | *-* |
| 1 condition only | 326 (21.2) | 262 (22.1) | 64 (18.3) | 0.23 (0.61 – 1.13) | 0.83 |
| 2 conditions only | 128 (7.9) | 94 (7.9) | 34 (9.7) | 1.22 (0.81 – 1.86) | 0.35 |
| 3 or more conditions | 100 (6.5) | 72 (6.1) | 28 (8.0) | 1.32 (0.83 – 2.09) | 0.24 |
| No Hypertension | 1280 (83.3) | 987 (83.2) | 293 (83.7) | Ref | - |
| Hypertension | 256 (16.7) | 199 (16.8) | 57 (16.3) | 0.97 (0.70 – 1.33) | 0.83 |
| No HIV | 1460 (95.1) | 1130 (95.3) | 330 (94.3) | Ref | - |
| HIV | 76 (4.9) | 56 (4.7) | 20 (5.7) | 1.22 (0.72 – 2.07) | 0.45 |
| No TB | 1530 (99.6) | 1181 (99.6) | 349 (99.7) | Ref | - |
| TB | 6 (0.4) | 5 (0.4) | 1 (0.3) | 0.68 (0.08 – 5.81) | > 0.99††† |
| No Diabetes | 1468 (95.6) | 1131 (95.4) | 337 (96.3) | Ref | - |
| Diabetes | 68 (4.4) | 55 (4.6) | 13 (3.7) | 0.79 (0.43 – 1.47) | 0.46 |
| No Cancer | 1445 (94.1) | 1115 (94.0) | 330 (94.3) | Ref | - |
| Any 1 Cancer | 80 (5.2) | 64 (5.4) | 16 (4.6) | 0.85 (0.48 – 1.48) | 0.56 |
| No Musculoskeletal Disorder | 1355 (88.2) | 1053 (88.8) | 302 (86.3) | Ref | - |
| Any 1 Musculoskeletal Disorder | 133 (11.8) | 100 (11.2) | 33 (13.7) | 1.15 (0.76 – 1.74) | 0.51 |
| No Cardiovascular Disease | 1509 (98.2) | 1166 (98.3) | 343 (98.0) | Ref | - |
| Any 1 Cardiovascular Disease | 24 (1.6) | 18 (1.5) | 6 (1.7) | 1.13 (0.45 – 2.88) | 0.79 |
| No Mental Health | 1457 (94.9) | 1131 (95.4) | 326 (93.1) | Ref | - |
| Any 1 Mental Health | 67 (5.1) | 46 (0.05) | 21 (0.07) | 1.58 (0.93 – 2.69) | 0.09 |
| No Respiratory Disease | 1483 (96.5) | 1149 (96.9) | 334 (95.4) | Ref | - |
| Any 1 Respiratory Disease | 51 (3.3) | 36 (3.0) | 15 (4.3) | 1.43 (0.78 – 2.65) | 0.25 |
| No Gastrointestinal Disease | 1482 (96.5) | 1144 (96.5) | 338 (96.6) | Ref | - |
| Any 1 Gastrointestinal Disease | 53 (3.5) | 42 (3.5) | 11 (3.1) | 0.89 (0.45 – 1.74) | 0.73 |
| No Sight Impairment | 1520 (99.0) | 1173 (98.9) | 347 (99.1) | Ref | - |
| Any 1 Sight Impairment | 13 (0.8) | 10 (0.8) | 3 (0.9) | 1.01 (0.28 – 3.71) | > 0.99††† |

† OR and CI obtained at OpenEpi.com. †† Chi-square tests for independence. ††† Fisher’s exact. Condition groups: cancer (lung, esophagus, cervix, breast (including benign), liver, prostate, colorectal and fibroid tumors); cardiovascular disease (fibrillation, TIA, stroke, PAD, MI, CHF); musculoskeletal disorder (osteoporosis, arthritis, joint and back pain); mental health (depression, anxiety, bipolar); gastrointestinal disease (Crohn’s disease, ulcer); respiratory disease (COPD, asthma); sight impairment (cataract, glaucoma, retinal).
