# Supplementary material for: The South African Rea Phela Health Study: A randomized controlled trial of communication retention strategies
Source: PLoS One. 2018 May 24;13(5):e0196900. doi: 10.1371/journal.pone.0196900 (PMC5967788; doi:10.1371/journal.pone.0196900)
Supplement: S2 Table — (DOCX) [file pone.0196900.s003.docx]

1

|  |  | Response Outcome | |  | |
| --- | --- | --- | --- | --- | --- |
| Type of Intervention | Specific Intervention | Paper / Mail  (n=295) | Electronic / Online  (n=55) | Odds ratio (95% confidence interval)† | *P*-value†† |
| Messaging Content | Generic Messaging | 103 (34.9) | 29 (52.7) | Ref | - |
|  | Themed Messaging | 126 (42.7) | 25 (45.5) | 0.70 (0.39 – 1.3) | 0.25 |
| Delivery Method | SMS Only (DM1) | 80 (27.1) | 10 (18.2) | Ref | - |
|  | **SMS + Postal (DM2)** | **67 (22.7)** | **29 (52.7)** | **3.46 (1.57 – 7.62)** | **0.001*** |
|  | Participant Choice (DM3) | 82 (27.8) | 15 (27.3) | 1.46 (0.62 – 3.4) | 0.38 |

† OR and CI obtained at OpenEpi.com. †† Chi-square tests for independence. *Bonferroni corrected significance for multiple tests (2), p < 0.025. No differences found between Themed and Generic Messaging. No differences found between SMS Only and SMS + Postal; SMS Only and Participant Choice; or SMS + Postal and Participant Choice. Controls were not the reference group because they were not given the online survey return option.
