# Supplementary material for: The South African Rea Phela Health Study: A randomized controlled trial of communication retention strategies
Source: PLoS One. 2018 May 24;13(5):e0196900. doi: 10.1371/journal.pone.0196900 (PMC5967788; doi:10.1371/journal.pone.0196900)
Supplement: S3 Table — (DOCX) [file pone.0196900.s004.docx]

**S3 Table. Differences in response by email provided and method of survey return.**

|  | Response Outcome  (n=40) | | |  | |
| --- | --- | --- | --- | --- | --- |
|  | Postal Mail  (n=295) | Online  (n=55) | Odds ratio (95% confidence interval)† | | *P*-value†† |
| No email provided | 232 (78.6) | 18 (32.7) | Reference | | - |
| **Email Provided** | **63 (21.4)** | **37 (67.3)** | **7.57 (4.04 – 14.19)** | | **<0.001** |

† OR and CI obtained at OpenEpi.com. †† Chi-square test for independence.
