# Supplementary material for: The South African Rea Phela Health Study: A randomized controlled trial of communication retention strategies
Source: PLoS One. 2018 May 24;13(5):e0196900. doi: 10.1371/journal.pone.0196900 (PMC5967788; doi:10.1371/journal.pone.0196900)
Supplement: S4 Table — (DOCX) [file pone.0196900.s005.docx]

|  | Intent-to-Treat | Response Outcome | |  | |
| --- | --- | --- | --- | --- | --- |
| Length of Time to First Contact | Participants (n=1536) | Non-Responders  (n=1186) | Responders (n=350) | Odds ratio (95% confidence interval)† | *P*-value†† |
| 180 days or less | 720 (46.9) | 570 (48.1) | 150 (42.9) | Ref | *-* |
| 181 days or more | 816 (53.1) | 616 (51.9) | 200 (57.1) | 0.81 (0.64 – 1.03) | 0.09 |

† OR and CI obtained at OpenEpi.com. †† Chi-square tests for independence.
