# Supplementary material for: The South African Rea Phela Health Study: A randomized controlled trial of communication retention strategies
Source: PLoS One. 2018 May 24;13(5):e0196900. doi: 10.1371/journal.pone.0196900 (PMC5967788; doi:10.1371/journal.pone.0196900)
Supplement: S5 Table — (DOCX) [file pone.0196900.s006.docx]

**S5 Table. Differences in response within interventions associated with response to first Madmaker.**

|  |  | Madmaker and Survey  Response Outcome  (n=40) | |  | |
| --- | --- | --- | --- | --- | --- |
| Type of Intervention | Specific Intervention | Non-responder  (n=25) | Responder  (n=15) | Odds ratio (95% confidence interval)† | *P*-value†† |
| Messaging Content | Generic Messaging | 16 (64.0) | 4 (26.7) | Ref | - |
|  | **Themed Messaging** | **9 (36.0)** | **11 (73.3)** | **4.89 (1.20 – 19.94)** | **0.048** |
| Delivery Method | SMS Only (DM1) | 4 (16.0) | 3 (20.0) | Ref | - |
|  | SMS + Postal (DM2) | 3 (12.0) | 6 (40.0) | 2.67 (0.35 – 20.51) | 0.66 |
|  | Participant Choice (DM3) | 18 (72.0) | 6 (40.0) | 0.44 (0.08 – 2.58) | 0.64 |

† OR and CI obtained at OpenEpi.com. †† Fisher’s exact. No statistical differences were found between delivery methods.
